# Effect of subcutaneous foslevodopa/foscarbidopa therapy on non-motor symptoms in advanced PD patients

**DOI:** 10.1007/s00415-026-13890-2

**Published:** 2026-06-08

**Authors:** Alina Jander, Sarah Bergner, Beate Schönwald, Monika Pötter-Nerger, Carsten Buhmann, Ute Hidding

**Affiliations:** Department of Neurology, University Medical Center Hamburg-Eppendorf, Martinistrasse 52, 20246 Hamburg, Germany

**Keywords:** Parkinson’s disease, Non-motor symptoms, Foslevodopa, Day clinic, Outpatient

## Abstract

**Background:**

Subcutaneous foslevodopa/foscarbidopa (LDp/CDp) infusion is approved for treating motor fluctuations and dyskinesias in patients with advanced Parkinson’s disease (aPD). However, efficacy data on non-motor symptoms (NMS) are limited.

**Objectives:**

To evaluate the effects of LDp/CDp treatment on NMS in patients with aPD.

**Design:**

Retrospective analysis of efficacy of LDp/CDp on NMS after 3 week therapy in a Parkinson’s day-clinic setting and further 6 weeks of outpatient treatment.

**Methods:**

Twenty-one patients with aPD were assessed at baseline (T0), after 3 weeks (T1), and after 9 weeks (T2). Assessments included broader NMS according to MDS-UPDRS Part I and NMS questionnaire (NMSQ) and in more detail sleep (PDSS-2) and depression (BDI-2).

**Results:**

Mean MDS-UPDRS I total score significantly improved by 27% at T1 and 34% at T2 (3.90 and 4.85 points, respectively), which has to be considered clinically relevant (threshold -2.64 points). Relevant improvement accounted for 62% of patients at T1 and 57% at T2.

MDS-UPDRS part IB score also improved significantly at both timepoints by approximately 30%. The greatest benefits were observed in sleep disturbances, pain, and constipation. NMSQ score improved by 21% at T1 and 27% at T2, particularly within the gastrointestinal domain. PDSS-2 score improved by 27% at T1, and Beck Depression Inventory 2 score by 37% at T1; both showed numerical but non-significant trends at T2. Psychotic symptoms occurred as adverse effects in 4 of 21 patients (19%).

**Conclusion:**

In this exploratory retrospective cohort, LDp/CDp therapy improved clinically relevant NMS, though confirmation in larger controlled studies is needed.

## Introduction

Advanced Parkinson’s disease (aPD) is characterized by the occurrence of both motor- and non-motor symptoms due to widespread neurodegeneration in the substantia nigra, brainstem and cerebral cortex [1]. Non-motor symptoms (NMS) include hyposmia, sleep disorders, autonomic symptoms like constipation and urinary incontinence, sensory aspects like pain and neuropsychiatric aspects like depression, psychosis and cognitive dysfunction with the possible development of dementia [2]. With disease progression, severity and fluctuations of motor and non-motor symptoms increase compromising the patient’s quality of life and often leading to hospitalization [3]. A cue therapeutic principle in patients with aPD is to provide a continuous dopaminergic drug delivery to avoid variable and fluctuating stimulation of striatal dopamine receptors due to loss of dopaminergic neurons as well as gastrointestinal dysfunction resulting in unstable drug resorption [4]. Medical pumps such as intrajejunal infusion of levodopa-carbidopa intestinal gel had been proven to improve not only motor fluctuations, but also NMS and quality of life [5, 6].

Recently, the therapeutic spectrum has been broadened by foslevodopa/foscarbidopa (LDp/CDp) continuous subcutaneous infusion treatment for patients with aPD [7] leading to improvement of motor fluctuations and dyskinesias [8, 9]. Non-motor symptoms have not been the focus of previous trials. However, positive effects on sleep and nocturia were reported [8, 10], while hallucinations and skin reactions were one of the most common reasons for discontinuing treatment [9, 11]. In the current study, we aimed to explore the effect of LDp/CDp on a broader spectrum of NMS in aPD patients 3 weeks after treatment initiation in the therapeutic setting of the Hamburg Parkinson’s day-clinic (HPDC) and further 6 week treatment in an outpatient setting.

## Patients and methods

### Patients

We included all patients with aPD and insufficiently controlled motor symptoms (i.e., motor fluctuations and/or dyskinesias) who had started continuous subcutaneous therapy with LDp/CDp at the Parkinson’s day-clinic of the Neurologic Department of the University Hospital Eppendorf (Hamburg, Germany) from January 2024 to September 2025 and maintained therapy for at least 9 weeks (*n*=21). Data were collected prospectively as part of routine clinical care and analyzed retrospectively. In patients with deep brain stimulation (DBS), settings were not changed during the study period to avoid bias on study outcome results.

### Methods

All clinically routinely assessed parameters regarding non-motor symptoms (NMS) were analyzed longitudinally before LDp/CDp therapy initiation (T0), after a 3 week period in the HPDC (T1), and after further 6 weeks (i.e., 9 weeks after LDp/CDp treatment initiation, T2). For T2 assessment, patients were re-admitted for 1 day to the HPDC after spending 6 weeks in an outpatient setting.

A total of six day-clinic visits were pre-scheduled within the first 3 weeks with the possibility of additional visits if medically necessary. In addition to the introduction of the pump device and advice of proper handling for patients and their caregivers, patients participated in the individualized integrated day-clinic program consisting of therapies provided by an interdisciplinary medical and nursing team as well as speech, occupational, and physical therapists, as previously described by us [12, 13].

Total score and sub-items of the revised Movement Disorder Society Unified Parkinson’s Disease Rating Scale (MDS-UPDRS) part 1 [14] were analyzed as outcome parameters to evaluate patient’s non-motor experiences of daily living. The MDS-UPDRS part IA as rater-based scale assesses neuropsychiatric symptoms, i.e., (i) cognitive impairment, (ii) hallucinations and psychosis, (iii) depressed mood, (iv) anxious mood, (v) apathy and (vi) features of dopamine dysregulation syndrome. The MDS-UPDRS part IB reflects self-rated (i) nighttime sleep problems, (ii) daytime sleepiness, (iii) pain and other sensations, (iv) urinary problems, (v) constipation problems, (vi) lightheadedness on standing, and (vii) fatigue.

Additionally, the 30-item-containing self-rated non-motor-symptom questionnaire (NMSQ) [15] was used to get an overview on the broad spectrum of NMS, the Parkinson’s Disease Sleep Scale 2 (PDSS-2) [16] and the Beck Depression Inventory 2 (BDI-2) [17] as further self-rated scores to address sleep and depression in more detail.

The MDS-UPDRS part 4 was used to evaluate motor fluctuations [14].

The different scales were used as part of standard clinical practice. All outcome assessments—including MDS-UPDRS 1B, NMSQ, PDSS-2, and BDI-2—were administered as standard components of the HPDC clinical protocol and were not modified or supplemented for the purpose of this analysis. MDS-UPDRS Part 1 and NMSQ were used in combination due to their complementary roles: the former provides a quantitative severity score with established MCID thresholds across a defined NMS domain set, while the latter captures a broader spectrum of 30 NMS as a binary prevalence checklist including domains not covered by MDS-UPDRS Part 1. PDSS-2 and BDI-2 were added to characterize sleep and depression in greater depth than single-item UPDRS subscores allow.

### Statistical analysis

Continuous normally distributed variables (age, disease duration, Hoehn and Yahr stage, MoCA) are presented as mean ± SD. Clinical scale scores which showed non-normal distributions are presented as median ± IQR. Statistical analyses were either performed by nonparametric Friedman test with pairwise comparison and Dunn’s correction or by mixed-effects analysis with Šídák's multiple comparisons test for three or more conditions. For analysis of subscores, mixed-effects analysis with Dunnett’s multiple comparison analysis was performed. Given the exploratory nature of this study and the small sample size, no correction for multiple comparisons across outcome domains was applied. Results should be interpreted as hypotheses-generating and require confirmation in larger controlled trials. To explore the association between improvements of non-motor symptoms with motor symptom amelioration or increased levodopa equivalent daily dose (LEDD), we calculated Spearman’s rank correlation coefficients (ρ). For each patient, changes in LEDD, MDS-UPDRS I, and MDS-UPDRS IV were computed as the difference between T0 and T2 values (indicated as Δ of LEDD, UPDRS I, and UPDRS IV respectively). *P* < 0.05 indicates statistical significance.

Statistical analyses were performed with GraphPad Prism (version 9.5.1 for Windows; GraphPad Software, LLC).

### Ethics

The study is a retrospective analysis of clinical data, conducted in accordance with the Declaration of Helsinki. According to the local ethics committee of Hamburg, there are no objections to publish this kind of data (reference number PV5799, WF-028/18).

## Results

A total of 21 patients (10 males, 5 with DBS) with aPD were analyzed. Patient characteristics before LDp/CDp therapy initiation are summarized in Table 1. Briefly, mean age was 70 ± 12 years; disease duration was 12 ± 6.3 years; mean Hoehn and Yahr stage was 2 ± 0.6. Mean Montreal Cognitive Assessment (MoCA) score was 25.7 ± 3.8. Mean LEDD before LDp/CDp was 1061 ± 419.8 mg. Mean LEDD at T2 was 1613 ± 636.4. Fourteen out of twenty-one patients were taking dopamine agonists prior to the initiation of pump therapy. These were discontinued in five patients, reduced in four patients, and continued unchanged in four patients.

**Table 1:** Patients baseline characteristics

Patient	Sex	Age, *y*	Disease duration, *y*	Hoehn and Yahr	MoCA (ON)	Pre LEDD	Dopamine agonists T0	Dopamine agonists T2	DBS
1	F	80	16	4	18	441	–	–	No
2	M	66	12	3	30	1115	8 mg rotigotine	2 mg rotigotine	Yes
3	F	89	5	2,5	24	1196	–	–	No
4	F	61	10	2	20	1523	4 mg ropinirole 8 mg rotigotine	–	No
5	F	65	16	2	21	563	–	–	Yes
6	M	67	11	2	26	1499	4 mg rotigotine	2 mg rotigotine	No
7	M	77	21	3	22	1490	2 mg rotigotine	-	No
8	F	80	23	2	27	1305	1,05 mg pramipexole	1,05 mg pramipexole	Yes
9	M	88	18	3	–	791	–	–	No
10	M	81	18	2	23	826	8 mg ropinirole	8 mg ropinirole	No
11	F	72	10	2	23	1685	8 mg ropinirole	–	No
12	M	70	4	3	27	1197	–	–	No
13	F	71	8	2	25	1105	4 mg ropinirole	4 mg ropinirole	No
14	M	62	16	2	30	965	10 mg rotigotine	6 mg rotigotine	Yes
15	M	77	23	2	24	861	4 mg rotigotine	2 mg rotigotine	No
16	F	57	9	2	30	1491	–	–	Yes
17	F	47	7	2	30	751	8 mg ropinirole	8 mg ropinirole	No
18	F	52	12	2	30	1000	–	–	
19	M	82	13	3	25	935	2 mg rotigotine	2 mg rotigotine	No
20	F	51	5	3	29	1328,5	250 mg piribedil	–	No
21	M	74	1	3	30	240	8 mg rotigotine	–	No
Mean ± SD		70 ± 12	12 ± 6.3	2 ± 0.6	25.7 ± 3.8	1061 ± 419.8			

Characteristics of all patients included (*n*=21). Values are given as mean (SD). *y* = years. Disease duration is calculated from symptom onset to initial pump device instrumentation. MoCA was assessed in MED and STIM ON. Pre LEDD (in mg) depicts the sum of all levodopa-containing and dopaminergic concomitant medication before LDp/CDp treatment initiation (T0)

*DBS* deep brain stimulation

As we have previously shown, motor efficacy assessed in our HPDC was comparable to results from clinical trials with significant improvement of motor fluctuations and dyskinesias with ameliorated quality of life [13]. In the cohort described there, the UPDRS IV score improved from median 8.5 (6–11.75) at T0 to median 3.5 (0–8.75) at T2 (*p*=0.0267) [13].

### MDS unified Parkinson’s disease rating scale part I (MDS-UPDRS I)

**Fig. 1:**
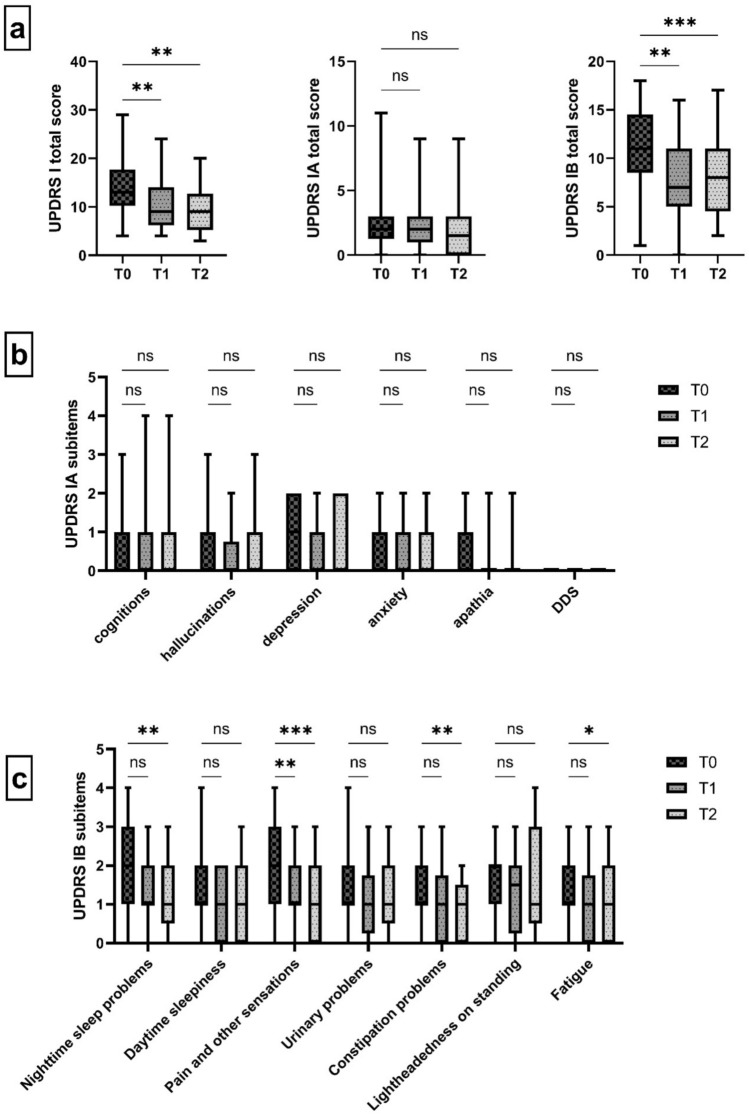
**a** Total score of UPDRS IA “non-motor aspects of experiences of daily living” (administered by rater), UPDRS IB “non-motor aspects of the experience of daily living” and complete UPDRS I at T0, T1 and T2. **b** Subscores of UPDRS IA at T0, T1, and T2. **c** Subscores of UPDRS IB at T0, T1, and T2. Data are presented as median ± interquartile range, and minimum/maximum. *n*= 21 at T0, T1, and T2, non-parametric Friedman test with Dunn’s multiple comparison analysis (A) and mixed-effects analysis with Dunnett's multiple comparison analysis (B+C). */**/*** = *p* < 0.05. *ns* not significant

In the present study, mean score of the total MDS-UPDRS I as parameter for non-motor aspects of daily living significantly improved after therapy initiation by 27 % (3.9 points) at T1 (T0 mean 14.25 ± 5.89, T1 mean 10.35 ± 5.3 (T0 vs. T1, *p* = 0.009)) and by 34 % (4.85 points) at T2 (T2 mean 9.4 ± 4.47 (T0 vs. T2, *p* = 0.002)). (Fig. 1a). Improvements at both time points must be considered as clinically meaningful as the Minimal Clinically Important Difference (MCID) threshold for changes has been described to be −2.64 points [18]. Improvement according to the MCID accounted for 62% (13/21) of patients at T1 and 57% (12/21) of patients at T2.

Mean score of the MDS-UPDRS IA as parameter for neuropsychiatric symptoms did not significantly change after 3 and 9 weeks of LDp/CDp therapy, but revealing a trend towards short-term and long-term improvement of 26% at both T1 and T2 (T0 mean 3.1 ± 2.75, T1 mean 2.3 ± 2.15 (T0 vs. T1, p 0.054) and T2 mean 2.3 ± 2.52 (T0 vs. T2, *p* = 0.18)). (Fig. 1a)

Mean score of the total MDS-UPDRS IB score prior to LDp/CDp therapy was 11.3 ± 4.2. Three weeks of LDp/CDp treatment in the HPDC lead to a significant reduction by 32 % to a mean score of 7.72 ± 4.33 (p = 0.0024) (T1). After an additional 6 weeks (T2), MDS-UPDRS IB remained stable with a mean score of 7.76 ± 4.1 (*p* = 0.0024), indicating improvement of a broader range of NMS. (Fig. 1a)

Assessment of the single sub-items of the MDS-UPDRS IA revealed no significant changes under LDp/CDp treatment in the short-term (T1) and long-term follow-up (T2) compared to T0; however, there were tendencies towards both short-term and long-term improvement. Cognition tended to be improved by 18% at T1 (T0 mean 0.67 ± 0.91, T1 mean 0.55 ± 1.1 (T0 vs. T1, *p*=0.88) and furthermore ameliorated by 22 % at T2 (T2 mean 0.52 ± 1.02 (T0 vs. T2, *p*=0.52)); depression tended to be reduced by 20 % at T1 (T0 mean 0.81 ± 0.87, T1 mean 0.65 ± 0.81 (T0 vs. T1, *p*=0.67)) and by 17 % at T2 (T2 mean 0.67 ± 0.93 (T0 vs. T2, *p*=0.52)). There was a tendency of reduction of anxiety by 19% at T1 (T0 mean 0.62 ± 0.80, T1 mean 0.5 ± 0.69 (T0 vs. T1, *p*=0.55)) and furthermore by 31% at T2 (T2 mean 0.43 ± 0.68 (T0 vs. T2, p = 0.32)). Apathy declined by 34 % at T1 (T0 mean 0.38 ± 0.59, T1 mean 0.25 ± 0.64 (T0 vs. T1, *p*=0.54)) and by 63 % at T2 (T2 mean 0.14 ± 0.48 (T0 vs. T2, *p*=0.18)), however not reaching the significance threshold. (Fig. 1b)

Assessment of the single subcategories of the MDS-UPDRS IB revealed a significant improvement in nighttime sleep disturbance with LDp/CDp treatment after 9 weeks. Mean value of nighttime sleep problems of 1.86 ± 1.24 at baseline was numerically reduced by 22% at T1 (1.45 ± 1.05; *p* = 0.10) and significantly reduced by 33 % at T2 compared to T0 (1.24 ± 0.89; *p* = 0.0097). Improvement of nighttime sleep problems got along by a trend with amelioration of daytime sleepiness by 37 % reduction at 3 weeks (mean 1.43 ± 0.98 at T0, mean 0.9 ± 0.91 at T1 (T0 vs. T1, *p* = 0.066)) and by 34 % reduction at 9 weeks follow-up (mean 0.95 ± 0.92 at T2 (T0 vs. T2, *p* = 0.0562)). Furthermore, numerical improvement of fatigue symptoms was observed with a 29 % reduction after 3 weeks (T0 mean 1.48 ± 1.03, T1 mean 1.05 ± 0.95 (T0 vs. T1 *p* = 0.1527)) with reaching significance by a 39 % reduction after 9 week follow-up (T2 mean 0.9 ± 0.94, T0 vs. T2 *p* = 0.0181)) (Fig. 1c).

Furthermore, the item “pain and other sensations” in the MDS-UPDRS I was significantly reduced by 37 % from mean 1.9 ± 1.14 at T0 to mean 1.2 ± 0.89 at T1 (*p* = 0.042) and by 42 % at T2 with a mean of 1.1 ± 0.94 (*p* = 0.0005). Constipation problems were significantly improved by 45 % after 9 weeks under LDp/CDp treatment (T0 mean 1.38 ± 0.87, T1 mean 1.05 ± 1.05 (24 % reduction, *p* = 0.2652), T2 mean 0.76 ± 0.83 (*p* = 0.0097)). Urinary problems were numerically improved under LDp/CDp treatment (T0 mean 1.57 ± 1.08, T1 mean 1.1 ± 0.91, T2 mean 1.29 ± 0.96). The item “lightheadedness on standing” did not change under LDp/CDp therapy implementation.

### NMS questionnaire (NMSQ)

**Fig. 2:**
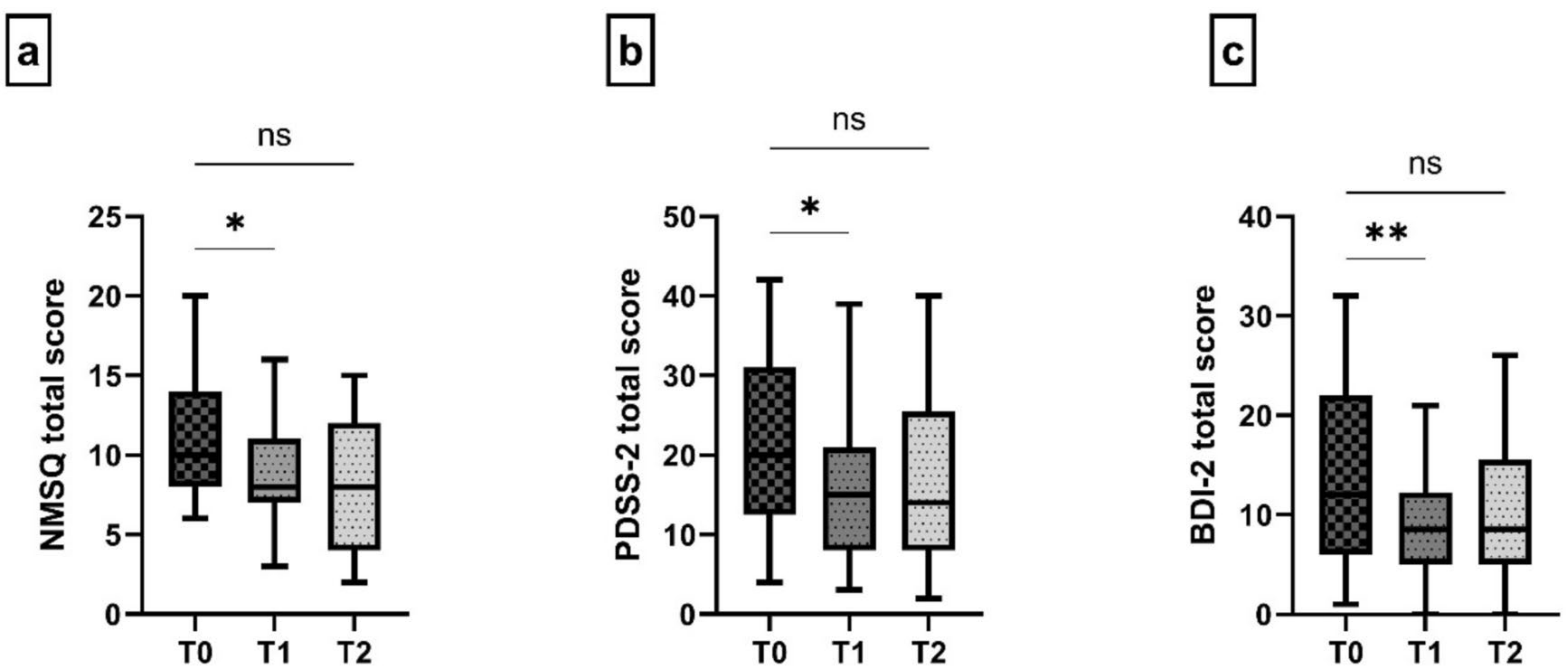
**a** Total score of NMS questionnaire with n=19 at all timepoints. **b** PDSS-2 total score at T0, T1 and T2. *n*=21 at T0, *n*=19 at T1 and *n*=21 at T2. **c** BDI-2 total score at T0, T1 and T2. **n**=19 at T0, **n**=18 at T1 and **n**=20 at T2. Data are presented as median ± interquartile range, and minimum/maximum. Non-parametric Friedman test with Dunn’s multiple comparison analysis (A), mixed-effects analysis with Šídák’s multiple comparisons test (B, C). */** = *p* < 0.05. *ns* not significant with *p* > 0.05

Nineteen patients completed the NMSQ for all time points. The mean score of non-motor symptoms on the NMSQ at T1 and T2 under LDp/CDp treatment significantly improved with a 21 % reduction at T1, maintained by a 27 % non-significant reduction at T2 compared to T0 (T0 mean 11.16 ± 3.93, T1 mean 8.84 ± 3.47 (T0 vs. T1 *p* = 0.0463), T2 mean 8.11 ± 3.97 (T0 vs. T2 *p* = 0.069)). (Fig. 2a)

Analysis of the sub-items (total number of “yes” answers) of the NMSQ after 3 and 9 week follow-up showed a numerical improvement for the items in the ‘gastrointestinal tract’ domain (total of 53 ‘yes’ answers at baseline to 37 at T1 and 22 at T2). This was mainly due to a reduction in dribbling, salivation, taste/smelling and swallowing, as well as constipation and incomplete emptying of the bowel. Furthermore, the domain ‘urinary tract’ numerically improved from 32 at T0 to 24 at T1 and 23 at T2, with both sub-items urgency and nocturia improved to the same extent. Regarding the cardiovascular system, there were no changes over time either in the overall domain or regarding the sub-items dizziness or edema. With respect to psychotic symptoms, hallucinations were numerically stable (5 ‘yes’ at baseline, 1 at T2, 5 at T2 with one patient with de novo hallucinations), but reports of delusions increased over the long term (1 ‘yes’ at baseline, 1 at T1, 4 at T2 with 3 de novo delusions). The category ‘apathy, attention, memory’ also showed short-term as well as long-term numerical improvement (T0: 24, T1: 16, T2: 18), mainly due to improved concentration and less loss of interest. Depression and anxiety also ameliorated both at T1 (13) and T2 (10) compared to T0 (17), with sub-item ‘sadness’ almost halved in the long-term evaluation (12 ‘yes’ at baseline, 10 at T1, 7 at T2). There was a positive effect on sleep and fatigue symptoms, with a clear reduction of sleep problems in the 9-week follow-up (T0 33 ‘yes’, T1: 32, T2: 23). There were no changes under LDp/CDp in the domain ‘miscellaneous’ (Table 2).

**Table 2:** Sub-items NMSQ: Number of patients who answered ‘yes’to each item of the NMS questionnaire and number of ‘yes’ answers in each domain (out of a total of 19 patients whocompleted the NMS questionnaire at all time points).

Domain	Item	Baseline	T1 (3 weeks)	T2 (9 weeks)
*Gastrointestinal tract*	*Total*	*53*	*37*	*22*
	Dribbling	8	5	4
	Taste/smelling	13	7	4
	Swallowing	9	4	3
	Vomiting	3	2	2
	Constipation	10	9	5
	Bowel incontinence	2	1	1
	Bowel emptying incomplete	8	9	3
*Urinary tract*	*Total*	*32*	*24*	*23*
	Urgency	15	11	11
	Nocturia	17	13	12
*Sexual function*	Total	10	7	10
	Sex drive	3	3	3
	Sex difficulty	7	4	7
*Cardiovascular*	*Total*	*11*	*16*	*12*
	Dizziness	7	10	7
	Swelling	4	6	5
*Apathy/attention/memory*	*Total*	*24*	*16*	*18*
	Remembering	6	5	5
	Concentrating	11	8	9
	Loss of interest	7	3	4
*Hallucinations/delusions*	*Total*	*6*	*2*	*9*
	Hallucinations	5	1	5
	Delusions	1	1	4
*Depression/anxiety*	*Total*	*17*	*13*	*10*
	Sadness	12	10	7
	Anxiety	5	3	3
*Sleep/fatigue*	*Total*	*33*	*32*	*23*
	Daytime sleepiness	3	3	1
	Insomnia	9	9	6
	Intense vivid dreams	6	6	5
	Acting out during dreams	5	6	4
	Restless legs	10	8	7
*Miscellaneous*	*Total*	*26*	*21*	*28*
	Weight	5	4	5
	Sweating	5	5	6
	Falling	8	6	8
	Pain	3	1	4
	Diplopia	5	5	5

### Parkinson disease sleep scale (PDSS-2)

Sleep disorders according to the Parkinson Disease Sleep Scale (PDSS-2) improved significantly by 28 % after 3 weeks from mean 21.52 ± 10.92 at T0 to mean 15.58 ± 9.26 points at T1 (*p* = 0.046). The short-term effect of LDp/CDp treatment on sleep disturbance was maintained after 9 weeks, however not reaching statistical significance (mean 16.81 ± 10.61 at T2, *p* = 0.214). (Fig. 2b)

### Beck depression inventory 2 (BDI-2)

After initiation of LDp/CDp treatment, depression assessed with the BDI-2 questionnaire was improved significantly already in the short-term follow-up by a 37 % reduction after 3 weeks (T0 mean 13.42 ± 8.51, T1 mean 8.44 ± 5.15 (T0 vs. T1 *p* = 0.0047)) and improvement was maintained at 9 week follow-up without reaching statistical significance (T2 mean 10.15 ± 7.23 (T0 vs. T2 *p* = 0.1008)) (Fig. 2c).

**Fig. 3:**
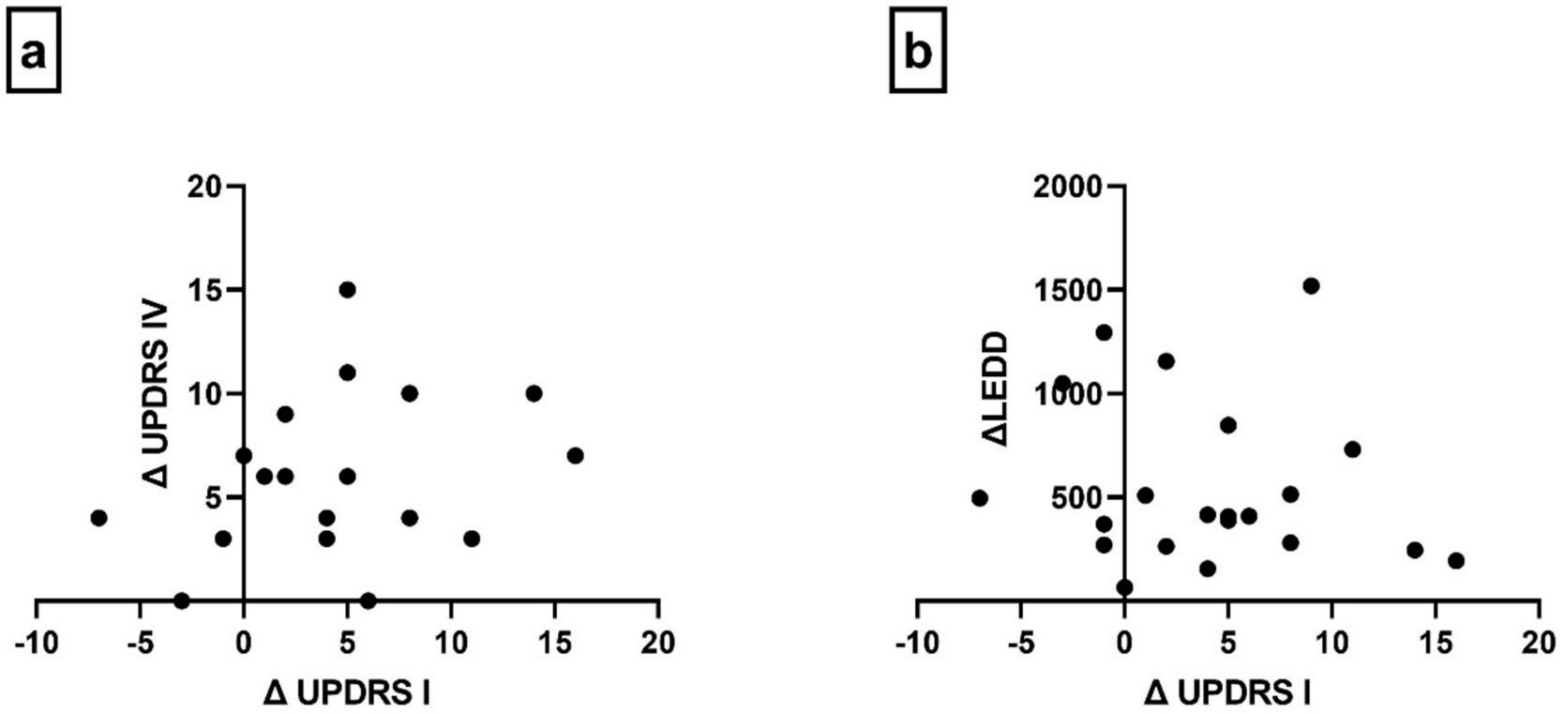
Scatter plots show the correlation between the change between T0 and T2 in UPDRS I total scores (Δ UPDRS I) and (**a**) the change in UPDRS IV scores (Δ UPDRS IV), and (**b**) the change in LEDD (Δ LEDD). Spearman’s rank correlation coefficient (Spearman r) and two-tailed *p* values were calculated. *n*=21 per plot

We correlated the improvement in UPDRS I total score with the improvement in UPDRS IV scores and the change in LEDD using Spearman’s rank correlation coefficients (*ρ*) (Fig. 3). There was neither a positive correlation between the changes in total UDPRS I total score and UPDRS IV (*ρ*=0.3, *p*=0.18) (Fig. 3a) nor between the changes in UPDRS I total score and the LEDD (*ρ*=0.1, *p*=0.65) (Fig. 3b).

## Discussion

In this study, we present clinical data showing that after initiation of subcutaneous LDp/CDp therapy in advanced PD in a day-clinic setting non-motor symptoms (NMS) according to MDS-UPDRS I total score, MDS-UPDRS IB score improved significantly after short-term follow-up at 3 weeks and this significance was maintained at 9 weeks under daily life conditions in an outpatient setting. NMSQ also improved significantly at T1, with a numerical but non-significant trend at T2. Among the NMS, sleep, pain, and gastrointestinal dysfunction showed the greatest numerical improvements, particularly at 9 weeks. Depression according to BDI-2 improved significantly at short-term follow-up after 3 weeks but did not maintain significance at 9 weeks, nor did PDSS-2.

While available studies primarily aimed to investigate improvement of motor fluctuations and dyskinesias with LDp/CDp treatment in patients with aPD [9, 11, 19], the effects on non-motor symptoms (NMS) are less well studied. However, some data suggest improvement of quality of life, sleep, and nocturia [8–11]. Non-motor fluctuations and their significant impact on the quality of life have recently been the focus of increased attention [20]. Continuous dopaminergic stimulation not only smoothes motor but likely also non-motor fluctuations. Accordingly, improvement of a broader range of NMS has been shown with respect to continuous subcutaneous apomorphine and intrajejunal levodopa infusion [21]. Therefore, subcutaneous LDp/CDp therapy might have similar effects, but systematic data addressing this hypothesis are lacking.

In our analysis, the MDS-UPDRS I showed an enhancement in non-motor symptoms after 9 weeks of LDp/CDp treatment, which can be attributed to an improvement in MDS-UPDRS IB. This improvement was driven by less self-reported problems with sleep disturbance, pain, and constipation. These results were even reflected in the NMSQ, which is a binary scale and therefore may be less sensitive to changes. Overall, the observed differences in total UPDRS I indicate clinically meaningful changes according to the suggested MCID thresholds (−2.64 for improvement in the MDS-UPDRS I) [18] and accounted for the majority of patients at both timepoints, T1 (62%; 13/21 patients) and T2 (57%; 12/21 patients).

Not all non-motor symptoms improved to the same extent and some symptoms even worsened. Therefore, total scores are only of limited significance, and we discuss individual symptoms or systems separately below.

### Sleep

Several reasons can contribute to sleep disturbances in PD. Neurodegeneration in sleep-regulating centers disturbs sleep [22], including REM sleep behavior disorder as non-motor sign very early in the course of the disease [23]. Non-motor symptoms such as bladder dysfunction, pain, or psychotic symptoms also frequently affect sleep [24]. Also sleep disorder due to motor impairment is a major problem in patients with aPD, presenting with nocturnal akinesia, OFF dystonia, or tremor. Especially these motor symptoms can be relieved by 24 h LDp/CDp infusion providing with continuous dopaminergic supply resulting in better sleep as has been showed by Chaudhuri et al. [8] and Aldred et al. [11] applying the PDSS-2 scale. Our data also show a significant improvement in the MDS-UPDRS IB nighttime sleep problems sub-item at T2 and in the PDSS-2 scale at T1 with a numerical, but not significant trend at T2. The sleep items in the NMSQ numerically improved either confirming the beneficial effect of LDp/CDp infusion on sleep. In addition to the pump’s hypothesized prevention of nocturnal dopamine level drops by continuous infusion, increased LEDD and the reduction of dopamine agonists represent potential contributing factors to sleep improvement; however, our observational data do not allow us to disentangle these effects.

### Hallucination/delusions

While levodopa carries a lower risk for psychosis than dopamine agonists, it can still lead to hallucinations, particularly in elderly or cognitively impaired patients with aPD. In the 12 month open-label safety study by Aldred et al., approximately 17 % of the patients suffered from hallucinations and 2.5 % from psychotic disorder under LDp/CDp treatment [11].

In our collective, hallucinations did not change significantly after 3 and 9 weeks according to MDS-UPDRS I and NMS questionnaire. However, the number of patients reporting delusional symptoms on the NMSQ increased markedly after 9 weeks of LDp/CDp therapy. Our findings are, therefore, in accordance with results from Aldred et al. A recent report by Brohée et al. even stated the development of delusions in five of nine patients after LDp/CDp treatment initiation [25]. Therefore, psychotic symptoms may occur not only acutely, but still after a longer period under LDp/CDp and should be assessed. A limitation of our analysis is that patients who discontinued therapy due to psychotic symptoms were not included. Therefore, data on the frequency of psychotic symptoms only refer to patients who used pump therapy for at least 9 weeks.

### Gastrointestinal dysfunction

Gastrointestinal dysfunction can occur at any stage of PD and can affect the entire length of the gastrointestinal tract [2]. The causes are multifactorial and include dysfunction of the enteric nervous system and the dorsal vagal motor nucleus, as well as axial rigidity and bradykinesia [26]. The effect of LDp/CDp on gastrointestinal dysfunction has not been studied yet

The following aspects are based on assumptions that are plausible from a pathophysiological perspective; however, they must be regarded as hypotheses, as they are not directly supported by data from this study. Results from our NMSQ evaluation suggest a numeric improvement in salivation and swallowing, possibly related to improved motor function of the pharyngeal muscles. Of note, improvement of defecation was self-reported in both the NMSQ and MDS-UPDRS I with less problems with constipation and incomplete evacuation of the bowel. These findings are consistent with those regarding jejunal levodopa administration [27]. As nigrostriatal dopaminergic dysfunction has been found to correlate with gastrointestinal dysautonomia in PD [28], one speculative hypothesis is that continuous levodopa delivery might benefit bowel function. Furthermore, along with the neurodegenerative processes, a range of barriers to levodopa transport and absorption such as dysphagia, delayed gastric emptying, constipation, *Helicobacter pylori* infection, small intestinal bacterial overgrowth and gut dysbiosis play a role in the pathogenesis of motor and non-motor fluctuations in PD [29]. Treatment with subcutaneous LDp/CDp bypasses the dysfunctional gastrointestinal (GI) tract and might therefore improve constipation as non-motor symptom itself. Another hypothesis is that replacing oral dopaminergic medication with subcutaneous delivery might reduce drug-related gastrointestinal effects due to transport and absorption issues [30].

We furthermore found a numerical improvement in smell and taste. Explanation is challenging, because hyposmia is primarily related to neurodegeneration in the olfactory bulb [31]. Indirect effects of subcutaneous LDp/CDp treatment such as a divergent perception due to diminished oral medication may play a role. The mechanism underlying any perceived improvement in smell and taste remains speculative; no objective olfactory assessment was performed.

### Pain

Pain is a common and etiologically diverse symptom in aPD patients [32]. There was a significant improvement in the “Pain and other sensations” item of the MDS-UPDRS I at 3 and 9 weeks which was not reflected in the NMSQ possibly due to its binary scale, which is less effective at capturing changes. In principle, pain in aPD patients occurs more frequently during motor OFF phases [33], so it seems plausible that a reduction in motor fluctuations is associated with an improvement in pain, though this remains unconfirmed in the present observational dataset.

### Further non-motor symptoms

We found no clear effect of LDp/CDp infusion on other non-motor symptoms, although the aspects sadness and bladder dysfunction were numerically self-reported to be slightly improved.

### Time course of efficacy on non-motor symptoms

The significant effects of LDp/CDp in the MDS-UPDRS IB were seen for the total score and the sub-item pain already after 3 weeks, but only after 9 weeks for sleep, constipation, and fatigue. However, for several outcome measures—including NMSQ, PDSS-2 and BDI-2—the statistically significant effect observed at T1 was not maintained at T2, despite persistent numerical improvement. This pattern suggests that either the initial effects partially waned on some domains over time, or that the study was underpowered to detect a statistically significant effect at the 9 week timepoint with this sample size. These findings should be interpreted with caution in the context of this exploratory cohort.

This improvement in non-motor symptoms resembles the observed motor improvement of ON and OFF times in the two pivotal studies, which showed significant improvements as early as 1 week after therapy initiation [9, 11]. Improvement increased within the following weeks, reaching a stable plateau after 4 weeks in the Soileau et al. study and after 12 weeks in the Aldred et al. study.

### Correlation between non-motor and motor symptoms and LEDD

An increase in LEDD, independent of subcutaneous administration, could conceivably account for the improvement in non-motor symptoms. In our cohort, however, no association was observed between changes in LEDD and non-motor outcomes; while this does not support a simple dose-dependent effect, we cannot conclude from these data alone that continuous or nighttime levodopa delivery is the driving mechanism, as other confounders may be responsible. Furthermore, the null correlation must be interpreted cautiously given the small sample size and the wide variance in both LEDD changes and NMS trajectories. Consistent with earlier reports [9, 11], motor fluctuations improved significantly in our study, as reflected by reduction in the MDS-UPDRS IV score. Yet these motor improvements did not correlate with changes in non-motor symptoms. Although motor and non-motor fluctuations frequently co-occur—for example, pain can be more prominent during motor OFF periods [34]—certain non-motor domains, such as nighttime sleep and gastrointestinal function, may improve independently of motor state. Furthermore, continuous subcutaneous LDp/CDp delivery may attenuate non-motor fluctuations by maintaining more stable plasma levodopa levels, thereby reducing the frequency and depth of non-motor OFF periods rather than—or in addition to—improving the baseline NMS symptoms themselves. These two mechanisms are not mutually exclusive, but they have different implications for prognosis: reduction of non-motor fluctuations would be expected to depend on continued therapy, while true baseline improvement in symptoms such as constipation might persist even during dose reduction. Finally, the concurrent increase in LEDD and the absence of a comparator group receiving equivalent doses of oral levodopa means we cannot exclude a general dose-dependent dopaminergic effect as a contributor to the observed NMS improvements, independent of the subcutaneous continuous delivery route.

### Limitations

Limitations include the small number of patients, the uncontrolled design, and the short observation period. The small sample size substantially limits statistical power and the reliability of subgroup analysis. Furthermore, results are only valid for patients who presented for the 9-week follow-up investigation and who undergone LDp/CDp therapy for at least 9 weeks. Patients who had previously discontinued treatment for any reason were not included, which represents a potential selection bias. Based on the study of a collective from a clinical setting, we did not include a control for placebo effect or a standard-of-care comparator. Thus, placebo effects cannot be ruled out. Furthermore, the observed improvements such as in MDS-UPDRS IB and PDSS-2 especially in the short-term period could partly be due to non-specific effects (e.g., the structured multidisciplinary care in the day-clinic, regression to the mean and medication adjustments such as reduction of dopamine agonists). Especially regarding the aspect of medication changes, it must be underlined at this point that dopamine agonists were reduced or discontinued in 9 of 21 patients which can substantially influence sleep and psychiatric symptoms (i.e., hallucinations, depression, impulse control disorder). Therefore, an analysis of subgroups would be advisable to attribute improvements to LDp/CDp treatment itself, however due to the small sample size in our investigation not reasonably conductible. Noteworthy, however, from a clinical point of view, reduction of dopamine agonists was only possible because of initiation of LDp/CDp therapy and therefore any improvement of patients due to less side effects of dopamine agonists are indirectly related to LDp/CDp treatment. Furthermore, the 9-week observation period is too short to draw conclusions about the long-term trajectory of non-motor symptoms. This is particularly relevant for depression, cognitive function, gastrointestinal dysfunction, and psychotic symptoms, which may show delayed emergence, relapse, or progression beyond the current study window. Specifically, psychiatric complications such as delusions may worsen over a longer treatment period, as suggested by the increasing rate observed at T2 in our cohort and corroborated by data from longer follow-up studies [11, 25]. Larger, randomized, and blinded studies are desirable to shed further light on these aspects.

## Conclusion

In conclusion, this exploratory retrospective study suggests that subcutaneous LDp/CDp therapy may improve certain non-motor symptoms in the short-term alongside its established motor benefits, with MDS-UPDRS I and IB showing sustained significance through 9 weeks. However, sleep, depression, and broader NMS were not statistically maintained in the long-term, and the small uncontrolled cohort limits the strength of these conclusions. Prospective, controlled studies with longer follow-up are warranted.

## Data Availability

The data sets generated during and/or analyzed during the current study are not publicly available due to privacy policy but are available from the corresponding author on reasonable request.

## Abbreviations

LDp/CDp: Subcutaneous foslevodopa/foscarbidopa
aPD: Advanced Parkinson’s disease
NMS: Non-motor symptoms
HPDC: Hamburg Parkinson’s day-clinic
NMSQ: Non-motor-symptom questionnaire
BDI-2: Beck depression inventory 2
MoCA: Montreal cognitive assessment
UPDRS: Unified Parkinson’s disease rating scale
MDS: Movement disorder society
PDSS-2: Parkinson disease sleep scale
DBS: Deep brain stimulation
LEDD: Levodopa equivalent daily dose
MCID: Minimal clinically important difference

## References

[1] Kalia LV, Lang AE (2015) Parkinson’s disease. Lancet 386:896–912. 10.1016/s0140-6736(14)61393-3 25904081 10.1016/S0140-6736(14)61393-3

[2] Schapira AHV, Chaudhuri KR, Jenner P (2017) Non-motor features of Parkinson disease. Nat Rev Neurosci 18(7):435–450. 10.1038/nrn.2017.62 28592904 10.1038/nrn.2017.62

[3] Classen J, Koschel J, Oehlwein C et al (2017) Nonmotor fluctuations: phenotypes, pathophysiology, management, and open issues. J Neural Transm 124:1029–1036. 10.1007/s00702-017-1757-0 28702850 10.1007/s00702-017-1757-0

[4] Bestetti A, Capozza A, Lacerenza M et al (2017) Delayed gastric emptying in advanced Parkinson disease: correlation with therapeutic doses. Clin Nucl Med 42:83–87. 10.1097/rlu.0000000000001470 27941374 10.1097/RLU.0000000000001470

[5] Kamel WA, Al-Hashel JY (2020) LCIG in treatment of non-motor symptoms in advanced Parkinson’s disease: review of literature. Brain Behav 10:e01757. 10.1002/brb3.1757 32677345 10.1002/brb3.1757 PMC7507541

[6] Tsunemi T, Oyama G, Saiki S et al (2021) Intrajejunal infusion of levodopa/carbidopa for advanced Parkinson’s disease: a systematic review. Mov Disord 36:1759–1771. 10.1002/mds.28595 33899262 10.1002/mds.28595 PMC9290931

[7] Rosebraugh M, Voight EA, Moussa EM et al (2021) Foslevodopa/Foscarbidopa: a new subcutaneous treatment for Parkinson’s disease. Ann Neurol 90(1):52–61. 10.1002/ana.26073 33772855 10.1002/ana.26073 PMC8251848

[8] Chaudhuri KR, Facheris MF, Bergmans B et al (2024) Improved sleep correlates with improved quality of life and motor symptoms with foslevodopa/foscarbidopa. Mov Disord Clin Pract 11:861–866. 10.1002/mdc3.14018 38465885 10.1002/mdc3.14018 PMC11233834

[9] Soileau MJ, Aldred J, Budur K et al (2022) Safety and efficacy of continuous subcutaneous foslevodopa-foscarbidopa in patients with advanced Parkinson’s disease: a randomised, double-blind, active-controlled, phase 3 trial. Lancet Neurol 21:1099–1109. 10.1016/s1474-4422(22)00400-8 36402160 10.1016/S1474-4422(22)00400-8

[10] Chaudhuri KR, Bouchard M, Freire-Alvarez E et al (2025) Post hoc exploratory analysis of the effect of foslevodopa/foscarbidopa continuous subcutaneous infusion on nocturia in patients with Parkinson’s disease. Clin Parkinsonism Relat Disord 12:100330. 10.1016/j.prdoa.2025.100330 10.1016/j.prdoa.2025.100330 PMC12099457 40417229

[11] Aldred J, Freire-Alvarez E, Amelin AV et al (2023) Continuous subcutaneous Foslevodopa/Foscarbidopa in Parkinson’s Disease: safety and efficacy results from a 12-month, single-arm, open-label, Phase 3 study. Neurol Ther 12(6):1937–1958. 10.1007/s40120-023-00533-1 37632656 10.1007/s40120-023-00533-1 PMC10630297

[12] Fründt O, Mainka T, Schönwald B et al (2018) The Hamburg Parkinson day-clinic: a new treatment concept at the border of in- and outpatient care. J Neural Transm 125:1461–1472. 10.1007/s00702-018-1918-9. (**20180822.**) 30167934 10.1007/s00702-018-1918-9

[13] Jander A, Bergner S, Schönwald B et al (2025) Subcutaneous foslevodopa/foscarbidopa initiation in a Parkinson’s day-clinic—a suitable setting to ensure treatment efficacy, tolerability and psychosocial adaption. Front Aging Neurosci 17:1619850. 10.3389/fnagi.2025.1619850. (**20250910.**) 41001152 10.3389/fnagi.2025.1619850 PMC12457366

[14] Goetz CG, Tilley BC, Shaftman SR et al (2008) Movement disorder society-sponsored revision of the Unified Parkinson’s disease rating scale (MDS-UPDRS): scale presentation and clinimetric testing results. Mov Disord 23:2129–2170. 10.1002/mds.22340 19025984 10.1002/mds.22340

[15] Chaudhuri KR, Martinez-Martin P, Schapira AH et al (2006) International multicenter pilot study of the first comprehensive self-completed nonmotor symptoms questionnaire for Parkinson’s disease: the NMSQuest study. Mov Disord 21:916–923. 10.1002/mds.20844 16547944 10.1002/mds.20844

[16] Trenkwalder C, Kohnen R, Högl B et al (2011) Parkinson’s disease sleep scale--validation of the revised version PDSS-2. Mov Disord 26:644–652. 10.1002/mds.23476. (**20110210.**) 21312275 10.1002/mds.23476

[17] Visser M, Leentjens AF, Marinus J et al (2006) Reliability and validity of the beck depression inventory in patients with Parkinson’s disease. Mov Disord 21:668–672. 10.1002/mds.20792 16450355 10.1002/mds.20792

[18] Horváth K, Aschermann Z, Kovács M et al (2017) Minimal clinically important differences for the experiences of daily living parts of movement disorder society-sponsored Unified Parkinson’s disease rating scale. Mov Disord 32:789–793. 10.1002/mds.26960. (**20170220.**) 28218413 10.1002/mds.26960

[19] Koeglsperger T, Berberovic E, Dresel C et al (2025) Real-world experience with continuous subcutaneous foslevodopa/foscarbidopa infusion: insights and recommendations. J Neural Transm. 10.1007/s00702-025-02911-5 40121314 10.1007/s00702-025-02911-5 PMC12855247

[20] Boura I, Poplawska-Domaszewicz K, Spanaki C et al (2025) Non-motor fluctuations in Parkinson’s disease: underdiagnosed yet important. J Mov Disord 18(1):1–16. 10.14802/jmd.24227 39703981 10.14802/jmd.24227 PMC11824532

[21] Mundt-Petersen U, Odin P (2017) Infusional therapies, continuous dopaminergic stimulation, and nonmotor symptoms. Int Rev Neurobiol 134:1019–1044. 10.1016/bs.irn.2017.05.036. (**20170724.**) 28805563 10.1016/bs.irn.2017.05.036

[22] Abbott SM, Videnovic A (2016) Chronic sleep disturbance and neural injury: links to neurodegenerative disease. Nat Sci Sleep 8:55–61. 10.2147/nss.S78947 26869817 10.2147/NSS.S78947 PMC4734786

[23] Berg D, Lang AE, Postuma RB et al (2013) Changing the research criteria for the diagnosis of Parkinson’s disease: obstacles and opportunities. Lancet Neurol 12:514–524. 10.1016/s1474-4422(13)70047-4 23582175 10.1016/S1474-4422(13)70047-4

[24] Rizos A, Martinez-Martin P, Odin P et al (2014) Characterizing motor and non-motor aspects of early-morning off periods in Parkinson’s disease: an international multicenter study. Parkinsonism Relat Disord 20:1231–1235. 10.1016/j.parkreldis.2014.09.013 25269446 10.1016/j.parkreldis.2014.09.013

[25] Brohée S, Roze E, Grabli D et al (2025) Cognitive and psychiatric adverse effects of foslevodopa/foscarbidopa in patients with Parkinson’s disease. Mov Disord Clin Pract. 10.1002/mdc3.70060 40152215 10.1002/mdc3.70060 PMC12275001

[26] Warnecke T, Schäfer KH, Claus I et al (2022) Gastrointestinal involvement in Parkinson’s disease: pathophysiology, diagnosis, and management. npj Parkinsons Dis 8:31. 10.1038/s41531-022-00295-x 35332158 10.1038/s41531-022-00295-x PMC8948218

[27] Honig H, Antonini A, Martinez-Martin P et al (2009) Intrajejunal levodopa infusion in Parkinson’s disease: a pilot multicenter study of effects on nonmotor symptoms and quality of life. Mov Disord 24:1468–1474. 10.1002/mds.22596 19425079 10.1002/mds.22596

[28] Hinkle JT, Perepezko K, Mills KA et al (2018) Dopamine transporter availability reflects gastrointestinal dysautonomia in early Parkinson disease. Parkinsonism Relat Disord 55:8–14. 10.1016/j.parkreldis.2018.08.010 30146185 10.1016/j.parkreldis.2018.08.010 PMC6291234

[29] Leta V, Klingelhoefer L, Longardner K et al (2023) Gastrointestinal barriers to levodopa transport and absorption in Parkinson’s disease. Eur J Neurol 30:1465–1480. 10.1111/ene.15734 36757008 10.1111/ene.15734

[30] Pagano G, Tan EE, Haider JM et al (2015) Constipation is reduced by beta-blockers and increased by dopaminergic medications in Parkinson’s disease. Parkinsonism Relat Disord 21(2):120–125. 10.1016/j.parkreldis.2014.11.015 25483722 10.1016/j.parkreldis.2014.11.015

[31] Wolters E, Braak H (2006) Parkinson’s disease: premotor clinico-pathological correlations. J Neural Transm Suppl. 10.1007/978-3-211-45295-0_47 17017546 10.1007/978-3-211-45295-0_47

[32] Mylius V, Perez Lloret S, Cury RG et al (2021) The Parkinson disease pain classification system: results from an international mechanism-based classification approach. Pain 162:1201–1210. 10.1097/j.pain.0000000000002107 33044395 10.1097/j.pain.0000000000002107 PMC7977616

[33] Storch A, Bremer A, Gandor F et al (2024) Pain fluctuations in Parkinson’s disease and their association with motor and non-motor fluctuations. J Parkinsons Dis 14:1451–1468. 10.3233/jpd-240026 39302380 10.3233/JPD-240026 PMC11492001

[34] Nebe A, Ebersbach G (2009) Pain intensity on and off levodopa in patients with Parkinson’s disease. Mov Disord 24:1233–1237. 10.1002/mds.22546 19412949 10.1002/mds.22546

